# Heart failure and diabetes: Understanding the bidirectional relationship

**DOI:** 10.1097/MD.0000000000034906

**Published:** 2023-09-15

**Authors:** Chukwuka Elendu, Dependable C. Amaechi, Tochi C. Elendu, Manizha Ashna, Jennifer Ross-Comptis, Sandra O. Ansong, Emmanuel O. Egbunu, Geraldine C. Okafor, Klein A. Jingwa, Akinbayo A. Akintunde, Chidinma M. Ogah, Melissa O. Edeko, Ayodeji V. Ibitoye, Mojetoluwa O. Ogunseye, Chisom E. Alakwe-Ojimba, Eunice K. Omeludike, Chibuike A. Oguine, Rechner N. Afuh, Clinton A. Olawuni, Onyekachukwu R. Ekwem, Boluwatife A. Oyedele, Erica I. Pius, Mercy O. Asekhauno, John A. Ladele, Chinonso B. Okoro, Brandon C. Monika Pouekoua, Joseph S. Adenikinju, Chigozirim M. Agu-ben, Ooreofe Aborisade

**Affiliations:** a Federal University Teaching Hospital, Owerri, Nigeria; b Igbinedion University Okada, Nigeria; c Imo State University, Nigeria; d University of Ottawa, Canada; e Larkin Community Hospital, United States; f Catholic Hospital Battor, Ghana; g Federal Medical Centre Bida, Nigeria; h University of Nigeria Teaching Hospital, Enugu, Nigeria; i Kazan State Medical University, Russia; j Babcock University, Nigeria; k Nationa Pirogov Memorial Medical University, Vinnytsia, Ukraine; l Chukwuemeka Odumegwu Ojukwu University, Nigeria; m University of Port Harcourt, Nigeria; n The Manor Hospital, Oxford, United Kingdom; o General Hospital, Odan, Lagos; p Delta State University, Nigeria; q Federal medical center Owerri, Nigeria; r University of Bamenda, Cameroon; s Northwick Park Hospital London Northwest NHS Trust, Nigeria; t Kharkiv Nationa Medical University, Ukraine; u Olabisi Onabanjo University Teaching Hospital, Nigeria.

**Keywords:** bidirectional relationship, diabetes mellitus, GLP-1 receptor agonists, heart failure, SGLT2 inhibitors

## Abstract

Heart failure and diabetes mellitus are 2 common and closely intertwined chronic conditions that often coexist in individuals. The relationship between heart failure and diabetes is bidirectional, meaning that each condition can influence the development and progression of the other. Understanding this complex interplay is crucial for optimizing the management and outcomes of patients with these comorbidities. This review comprehensively analyzed the literature to examine the bidirectional relationship between heart failure and diabetes. We searched various electronic databases and included studies that explored the pathophysiological mechanisms, epidemiology, clinical implications, and therapeutic considerations associated with this relationship. The bidirectional relationship between heart failure and diabetes is multifactorial and involves several interconnected mechanisms. Diabetes is a recognized risk factor for heart failure, increasing the risk of its development and accelerating its progression. On the other hand, heart failure can contribute to the development of insulin resistance and worsen glycemic control in patients with diabetes. Shared risk factors, such as obesity, hypertension, and dyslipidemia, contribute to development of both conditions. Additionally, hyperglycemia, insulin resistance, chronic inflammation, oxidative stress, and mitochondrial dysfunction play significant roles in the pathogenesis of heart failure in individuals with diabetes. The bidirectional relationship between heart failure and diabetes has important clinical implications. Patients with heart failure and diabetes have worse outcomes, including higher hospitalization rates, morbidity, and mortality, than those without diabetes. Optimal management strategies should target both conditions simultaneously, focusing on lifestyle modifications, pharmacotherapy, glycemic control, and cardiovascular risk reduction.

## 1. Introduction and background

Heart failure (HF) and diabetes mellitus are prevalent chronic conditions with substantial global burdens. Both states significantly impact morbidity, mortality, and healthcare costs.^[1]^ What makes the relationship between HF and diabetes particularly noteworthy is its bidirectional nature, wherein each condition influences the development and progression of the other. Understanding the intricate interplay between HF and diabetes is crucial for optimizing patient management and improving outcomes in individuals with these comorbidities.

Diabetes mellitus, characterized by chronic hyperglycemia, affects approximately 9.3% of the global population.^[2]^ It is well-established that diabetes is a significant risk factor for the development of cardiovascular diseases, including HF. Several population-based studies have consistently demonstrated that diabetes increases the risk of HF by 2- to 5-fold.^[3,4]^ The underlying mechanisms linking diabetes and HF involve complex pathophysiological processes, including insulin resistance, endothelial dysfunction, inflammation, and oxidative stress.^[5]^

On the other hand, HF can also contribute to the development and worsening of glucose metabolism abnormalities, leading to the onset of diabetes or the deterioration of glycemic control in individuals with preexisting diabetes. This bidirectional relationship is influenced by shared risk factors such as obesity, hypertension, and dyslipidemia, as well as systemic inflammation and neurohormonal activation.^[6]^

Given the bidirectional nature of the relationship between HF and diabetes, it becomes essential to recognize and address both conditions simultaneously to achieve optimal outcomes. Integrated care models involving multidisciplinary teams, including cardiologists, endocrinologists, and primary care physicians, are essential for comprehensive management strategies for individuals with HF and diabetes.^[7]^

This review aims to analyze the bidirectional relationship between HF and diabetes comprehensively. Examining the existing literature will explore the pathophysiological mechanisms, epidemiology, clinical implications, and therapeutic considerations associated with this complex relationship.

## 2. Objectives of the study

To examine the pathophysiological mechanisms underlying the bidirectional relationship between HF and diabetes mellitus.

To assess the epidemiological characteristics and prevalence of the coexistence of HF and diabetes.

To explore the clinical implications of the bidirectional relationship, including the impact on morbidity, mortality, and healthcare utilization.

Identify the therapeutic considerations and management strategies for individuals with HF and diabetes.

To highlight the importance of integrated care models involving multidisciplinary teams in optimizing outcomes for patients with HF and diabetes.

To identify gaps in current knowledge and areas for future research regarding the bidirectional relationship between HF and diabetes.

## 3. Review

### 3.1. Methodology

This review comprehensively analyzed the literature to examine the bidirectional relationship between HF and diabetes mellitus. The methodology involved a systematic search of electronic databases, including PubMed, MEDLINE, and Google Scholar, using relevant keywords such as “HF,” “diabetes mellitus,” “bidirectional relationship,” and variations thereof. The search covered a time range from the databases’ inception to the knowledge cutoff of September 2021.

The inclusion criteria for the selection of studies included the following:

Studies published in peer-reviewed journals.

Studies that investigated the bidirectional relationship between HF and diabetes.

Studies that examined the pathophysiological mechanisms, epidemiology, clinical implications, and therapeutic considerations associated with the relationship.

Studies published in English.

The initial search yielded many articles, and duplicates were removed. The remaining articles were screened based on their titles and abstracts for relevance to the research topic. Full-text articles were then retrieved for further evaluation.

The selected articles were critically reviewed and synthesized to extract relevant information about the study objectives. Data on pathophysiological mechanisms, epidemiology, clinical implications, and therapeutic considerations were extracted and analyzed. Key findings and trends were identified and organized under appropriate subheadings.

The study limitations include potential publication bias and the exclusion of non-English articles, which may have resulted in the omission of relevant studies. However, efforts were made to include a wide range of studies to provide a comprehensive analysis of the bidirectional relationship between HF and diabetes.

The findings and discussions presented in this review are based on the analysis and synthesis of the selected literature. The conclusions drawn are derived from the collective evidence available in the literature. They should be interpreted in the context of the study limitations and existing knowledge in the field.

### 3.2. Epidemiology

The bidirectional relationship between HF and diabetes mellitus has significant implications for the epidemiology of both conditions. The coexistence of HF and diabetes is common and has been extensively studied.

Epidemiological studies consistently demonstrate that diabetes is a significant risk factor for the development of HF. The prevalence of HF is significantly higher in individuals with diabetes compared to those without diabetes. It has been estimated that approximately 30% to 40% of patients with HF have concurrent diabetes.^[8]^ Moreover, diabetes increases the risk of incident HF by 2- to 5-fold.^[4,6]^ The risk appears higher in individuals with type 2 diabetes than those with type 1 diabetes.^[9]^ The relationship between diabetes and HF is observed across different populations and ethnicities.^[10]^

Conversely, HF can also contribute to the development and worsening of diabetes. Studies have shown that the prevalence of diabetes is higher among patients with HF compared to the general population.^[11]^ The presence of HF increases the risk of developing diabetes, and this risk is further amplified in patients with reduced ejection fraction and advanced HF.^[12]^ The mechanisms underlying the development of diabetes in HF patients involve factors such as insulin resistance, impaired glucose metabolism, and neurohormonal dysregulation.^[13]^

Shared risk factors play a crucial role in the epidemiology of HF and diabetes. Obesity, hypertension, dyslipidemia, and a sedentary lifestyle are common risk factors for both conditions.^[14]^ These risk factors contribute to the development and progression of HF and diabetes, establishing a bidirectional relationship between the 2.

The epidemiology of HF and diabetes has important implications for healthcare utilization and outcomes. The coexistence of HF and diabetes is associated with worse results, including increased hospitalization rates, morbidity, and mortality. Patients with HF and diabetes have higher healthcare costs and require more intensive management strategies than those without diabetes.^[15]^

In conclusion, the bidirectional relationship between HF and diabetes significantly impacts their epidemiology. Diabetes is a significant risk factor for the development of HF, while HF increases the risk of developing and worsening diabetes. Shared risk factors contribute to the coexistence of these conditions. Understanding the epidemiological aspects of HF and diabetes is crucial for effective management and prevention strategies.

### 3.3. Pathophysiology

The bidirectional relationship between HF and diabetes mellitus is complex and involves an intricate interplay between the molecular, cellular, and systemic conditions. Several pathophysiological mechanisms contribute to the development and progression of HF and diabetes, and understanding these mechanisms is crucial for comprehending their bidirectional association. The pathophysiology of the HF-diabetes relationship can be described as follows:

#### 3.3.1. Insulin resistance and hyperglycemia.

Insulin resistance, a hallmark of type 2 diabetes, is a central component of the pathophysiology linking HF and diabetes. In insulin resistance, tissues such as skeletal muscle, adipose tissue, and the liver exhibit reduced responsiveness to insulin, leading to impaired glucose uptake and utilization. Hyperglycemia ensues due to reduced glucose uptake in peripheral tissues and increased hepatic glucose production. Insulin resistance is common in both conditions and is influenced by shared risk factors, including obesity, sedentary lifestyle, and systemic inflammation.^[5]^

#### 3.3.2. Neurohormonal dysregulation.

HF and diabetes are characterized by dysregulation of neurohormonal pathways, particularly the sympathetic nervous system and the renin-angiotensin-aldosterone system. In HF, reduced cardiac output and impaired tissue perfusion trigger compensatory mechanisms, leading to sympathetic activation and increased catecholamine release. Chronic sympathetic stimulation contributes to insulin resistance and hyperglycemia in diabetes. Similarly, in diabetes, hyperglycemia and insulin resistance may stimulate sympathetic activity, exacerbating cardiovascular dysfunction and contributing to the development of HF.^[16]^

#### 3.3.4. Oxidative stress and inflammation.

Both HF and diabetes are associated with increased oxidative stress and systemic inflammation. Chronic inflammation and oxidative stress play a critical role in the pathogenesis of insulin resistance, impaired endothelial function, and vascular damage. In HF, oxidative stress contributes to myocardial dysfunction, fibrosis, and adverse remodeling, while in diabetes, it promotes endothelial dysfunction, atherosclerosis, and microvascular complications. Shared inflammatory pathways may contribute to the cross-talk between HF and diabetes.^[17,18]^

#### 3.3.5. Endothelial dysfunction.

Endothelial dysfunction, characterized by impaired nitric oxide bioavailability and increased endothelin-1 production, is a crucial factor in the pathogenesis of both HF and diabetes. Endothelial dysfunction contributes to vascular stiffness, impaired vasodilation, and microvascular abnormalities, leading to systemic and coronary microcirculation impairments. In HF, endothelial dysfunction may contribute to impaired cardiac contractility and coronary perfusion, while in diabetes, it contributes to atherosclerosis and diabetic cardiomyopathy.^[17]^

#### 3.3.6. Myocardial lipotoxicity and steatosis.

In diabetes, excessive circulating free fatty acids and triglycerides contribute to myocardial lipotoxicity and steatosis, impairing myocardial energetics and contractile dysfunction. Myocardial steatosis may promote cardiomyocyte apoptosis and fibrosis. In HF, elevated free fatty acids exacerbate myocardial lipotoxicity and oxidative stress, leading to further myocardial dysfunction and remodeling.^[16]^ These lipid-related mechanisms may contribute to the development of diabetic cardiomyopathy and the progression of HF.

#### 3.3.7. Diabetic cardiomyopathy.

Diabetic cardiomyopathy represents a unique form characterized by myocardial dysfunction in the absence of coronary artery disease or hypertension. The underlying pathophysiology involves a combination of myocardial fibrosis, impaired calcium handling, altered energetics, and inflammatory processes. Diabetic cardiomyopathy may predispose individuals to the development of HF.^[18]^

#### 3.3.8. Microvascular dysfunction.

Microvascular dysfunction is a shared pathophysiological mechanism in both HF and diabetes. In diabetes, impaired microvascular circulation contributes to diabetic nephropathy, retinopathy, and neuropathy. Similarly, microvascular dysfunction in HF may lead to impaired organ perfusion, renal dysfunction, and tissue hypoxia.^[17,18]^

#### 3.3.9. Shared risk factors.

HF and diabetes share several risk factors, such as obesity, hypertension, dyslipidemia, and a sedentary lifestyle. These risk factors contribute to insulin resistance, endothelial dysfunction, inflammation, and oxidative stress, linking the pathophysiological processes of both conditions.

The bidirectional relationship between HF and diabetes involves a complex interplay of shared risk factors, pathophysiological mechanisms, and molecular processes. The pathophysiology of this relationship highlights the importance of early detection, integrated management strategies, and targeted therapeutic approaches to address the comorbidities and improve outcomes in individuals affected by HF and diabetes.^[5,16–18]^

### 3.4. Laboratory abnormalities and diagnosis

Diagnosing and managing the bidirectional relationship between HF and diabetes mellitus require a comprehensive evaluation, including laboratory investigations. Several laboratory abnormalities are associated with both conditions and are crucial in their diagnosis and monitoring.

#### 3.4.1. Glycemic markers.

Diabetes is characterized by hyperglycemia, and the measurement of glycemic control is essential in diagnosing and managing the condition. Glycosylated hemoglobin levels indicate long-term glucose control, reflecting the average blood glucose levels over the past few months.^[19]^ In patients with HF, diabetes, or both conditions, monitoring Glycosylated hemoglobin levels can help assess glycemic control and guide treatment strategies to improve outcomes.

#### 3.4.2. Cardiac biomarkers.

In HF, cardiac biomarkers such as B-type natriuretic peptide and N-terminal pro-B-type natriuretic peptide are essential diagnostic tools. These biomarkers are elevated in response to cardiac stress and correlate with the severity of HF. In patients with diabetes and HF, assessing cardiac biomarkers can aid in diagnosing HF, monitoring disease progression, and guiding treatment decisions.^[20]^

#### 3.4.3. Lipid profile.

Dyslipidemia is a common risk factor for HF and diabetes. Measuring the lipid profile, including total cholesterol, low-density lipoprotein cholesterol, high-density lipoprotein cholesterol, and triglycerides, is crucial in identifying lipid abnormalities and addressing cardiovascular risk factors in patients with both conditions.^[21]^

#### 3.4.4. Kidney function tests.

Both HF and diabetes can lead to kidney dysfunction. Monitoring kidney function through blood tests, such as serum creatinine and estimated glomerular filtration rate, is essential to assess renal health and identify any deterioration in kidney function, which may impact treatment decisions and outcomes.^[22]^

#### 3.4.5. Electrolytes and fluid balance.

Electrolyte imbalances, particularly hypokalemia, and hyponatremia, are common in HF and may also occur in some diabetic patients. Regularly monitoring electrolyte levels is necessary to guide treatment and prevent complications related to imbalances.^[23]^

#### 3.4.6. Inflammatory markers.

Chronic inflammation plays a role in the pathophysiology of both HF and diabetes. Measurement of inflammatory markers, such as C-reactive protein and pro-inflammatory cytokines (e.g., interleukin-6), can provide insights into the inflammatory state and guide treatment strategies targeting inflammation.^[24]^

#### 3.4.7. Imaging and functional tests.

Echocardiography is a valuable imaging tool to assess cardiac structure and function in HF and diabetes patients. It provides information on left ventricular function, ejection fraction, and any structural abnormalities, aiding in the diagnosis and management of HF and assessing its impact on patients with diabetes.^[20]^

Diagnosing the bidirectional relationship between HF and diabetes requires a comprehensive evaluation integrating clinical assessment, laboratory abnormalities, and imaging findings. A multidisciplinary approach involving cardiologists, endocrinologists, and other specialists is crucial for accurate diagnosis, appropriate management, and individualized treatment plans.

### 3.5. Management

Managing the bidirectional relationship between HF and diabetes mellitus requires a comprehensive and multidisciplinary approach. The primary goals of management include controlling blood glucose levels, optimizing cardiac function, preventing complications, and improving the overall quality of life. Table 1 describes managing the bidirectional relationship between HF and diabetes mellitus.

**Table 1:** Management of the bidirectional relationship between HF and diabetes mellitus.

Lifestyle Modifications: Diet: Encourage a balanced diet rich in fruits, vegetables, whole grains, lean proteins, and healthy fats. Limit the intake of processed foods, sugary beverages, and saturated and trans fats. Physical Activity: Encourage regular aerobic exercise, such as brisk walking or cycling and resistance training, to improve cardiovascular fitness, glycemic control, and overall well-being. Weight Management: Promote weight loss or maintenance through calorie control and a combination of diet and exercise, aiming for healthy body weight. Smoking Cessation: Advise and support patients to quit smoking as it worsens HF and diabetes.^[19]^ Glycemic Control: Medications: Prescribe antihyperglycemic medications tailored to individual needs, considering glycemic targets, comorbidities, and potential cardiovascular effects. Options include metformin, sulfonylureas, dipeptidyl peptidase-4 (DPP-4) inhibitors, sodium-glucose cotransporter-2 (SGLT2) inhibitors, and glucagon-like peptide-1 (GLP-1) receptor agonists. Self-Monitoring: Encourage regular self-monitoring of blood glucose levels to assess glycemic control and adjust medication regimens. Education: Provide comprehensive diabetes self-management education, including instruction on blood glucose monitoring, medication administration, healthy eating, physical activity, and symptom recognition.^[25]^ Heart Failure Management: Medications: Optimize HF therapy based on guideline-directed medical treatment, including beta-blockers, angiotensin-converting enzyme inhibitors/angiotensin receptor blockers (ACE inhibitors/ARBs), mineralocorticoid receptor antagonists (MRAs), and diuretics. Consider using newer agents, such as angiotensin receptor-neprilysin inhibitors (ARNIs) and SGLT2 inhibitors, which have shown benefits in HF patients with or without diabetes. Device Therapy: Consider implantable cardioverter-defibrillators (ICDs) or cardiac resynchronization therapy (CRT) devices in eligible patients with reduced ejection fraction and HF to reduce mortality and improve symptoms. Fluid and Sodium Restriction: Advise patients to limit sodium intake and maintain fluid balance to manage HF symptoms and prevent fluid overload. Regular Follow-up: Schedule regular follow-up visits to monitor symptoms, optimize medications, and assess disease progression. Collaborate with a multidisciplinary team, including cardiologists, endocrinologists, and specialized nurses, to provide comprehensive care.^[19]^ Cardiovascular Risk Management: Lipid Management: Treat dyslipidemia with statins or other lipid-lowering agents to reduce cardiovascular risk. Aim for target LDL-C levels based on individual risk profiles. Blood Pressure Control: Aggressively manage hypertension using lifestyle modifications and antihypertensive medications to achieve target blood pressure goals. Antiplatelet Therapy: Prescribe antiplatelet therapy, such as low-dose aspirin, in patients with established cardiovascular disease while considering the balance between risks and benefits. Comprehensive Risk Factor Management: Address other modifiable risk factors, such as smoking cessation, weight management, and controlling comorbidities like kidney disease and atrial fibrillation.^[21]^ Patient Education and Support: Self-Care Management: Provide education and support to patients for self-care management of both HF and diabetes. This includes proper medication adherence, monitoring symptoms, and glucose levels, recognition of worsening HF or hyperglycemia, and adherence to lifestyle modifications. Disease Education: Educate patients about the nature of HF and diabetes, their interrelationship, and the importance of adherence to treatment plans and regular follow-up visits. Psychosocial Support: Address the emotional and psychological aspects of living with HF and diabetes through counseling, support groups, and referral to mental health professionals if needed. Caregiver Support: Involve caregivers in the management process, providing them with education and resources to support the patient care and overall well-being. Coordinated Care: Multidisciplinary Approach: Foster collaboration among healthcare professionals, including cardiologists, endocrinologists, primary care physicians, nurses, dietitians, and pharmacists, to ensure coordinated and comprehensive care. Care Transitions: Facilitate smooth transitions between care settings, such as hospital to home or primary care to specialist care, to prevent gaps in management and ensure continuity of care. Care Plans and Self-Management Tools: Develop individualized care plans that outline treatment goals, medications, lifestyle recommendations, and monitoring parameters. Provide patients with self-management tools, such as medication diaries, glucose monitoring devices, and symptom trackers, to empower them in their care. Regular Monitoring and Follow-up: Assessments: Regularly evaluate HF symptoms, glycemic control, cardiovascular risk factors, and medication adherence. Laboratory Monitoring: Monitor glycemic markers (e.g., FPG, HbA1c) and cardiac biomarkers (e.g., BNP, NT-proBNP) to assess disease control, treatment response, and the risk of complications. Imaging and Functional Tests: Perform periodic imaging tests, such as echocardiography and stress testing, to evaluate cardiac structure and function and monitor disease progression. Collaborative Management: Maintain open communication between healthcare providers and patients, ensuring that any concerns or changes in symptoms are addressed promptly. It is important to note that management strategies should be individualized based on patient characteristics, preferences, comorbidities, and disease severity. Regular reassessment and adjustment of management plans are necessary to optimize outcomes and improve patients’ overall health and well-being with the bidirectional relationship between HF and diabetes.

BNP = B-type natriuretic peptide, FPG = fasting plasma glucose, HbA1c = glycosylated hemoglobin, LDL-C = low-density lipoprotein cholesterol, NT-proBNP = N-terminal pro-B-type natriuretic peptide, SGLT2 = sodium-glucose co-transporter 2 inhibitor.

### 3.6. New York heart association classification

The classification for managing HF is typically based on the severity of symptoms and the degree of functional impairment.^[23]^ The New York Heart Association (NYHA) functional classification is the most commonly used classification system, categorizing HF into 4 classes.^[20]^ Although the NYHA classification may not directly relate to the bidirectional relationship between HF and diabetes, it plays a crucial role in the comprehensive evaluation and management of HF patients, including those with diabetes. The management approach may vary depending on the class of HF. Here is an overview of the NYHA classification for managing HF:

#### 3.6.1. Class I.

Patients with Class I HF have no limitations or symptoms of HF during ordinary physical activity. They do not experience shortness of breath, fatigue, or palpitations unless they exercise strenuously.^[23]^ The management goal for Class I HF is to prevent disease progression and optimize cardiovascular health through lifestyle modifications, risk factor management, and appropriate monitoring.^[26]^

#### 3.6.2. Class II.

Patients with Class II HF have mild limitations and symptoms of HF during ordinary physical activity. They may experience fatigue, shortness of breath, or palpitations with moderate exertion.^[23]^ The management goal for Class II HF is to relieve symptoms, improve exercise tolerance, and prevent hospitalization. It typically involves lifestyle modifications, medication therapy (such as diuretics, beta-blockers, and angiotensin-converting enzyme/angiotensin receptor blockers), and patient education on self-care management.^[27]^

#### 3.6.3. Class III.

Patients with Class III HF have marked limitations and symptoms of HF during less-than-ordinary physical activity. They may experience fatigue, shortness of breath, or palpitations with minimal exertion.^[28]^ The management goal for Class III HF is to stabilize symptoms, improve quality of life, and reduce the risk of hospitalization. Treatment strategies often include lifestyle modifications, optimized medication therapy, cardiac rehabilitation, and close monitoring.^[28]^

#### 3.6.4. Class IV.

Patients with Class IV HF have severe limitations and symptoms of HF at rest or with any physical activity. They may experience severe fatigue, shortness of breath, or palpitations even at rest.^[29]^ The management goal for Class IV HF is to alleviate symptoms, improve comfort, and prevent further deterioration.^[30]^ Treatment approaches may include advanced therapies such as heart transplantation, mechanical circulatory support, palliative care, and symptom management.

It important to note that the management of HF is individualized based on the patient specific clinical profile, comorbidities, and response to treatment. Regular follow-up visits, monitoring of symptoms and disease markers, and adjustments in management plans are necessary to optimize outcomes and improve the patient overall well-being.^[31]^

### 3.7. Prognosis

The prognosis of patients with the bidirectional relationship between HF and diabetes mellitus can be influenced by various factors, including the severity of HF, the degree of glycemic control, comorbidities, and adherence to treatment regimens.^[32]^ Here are some key points regarding the prognosis:

#### 3.7.1. Increased cardiovascular risk.

The presence of both HF and diabetes significantly increases the risk of cardiovascular events, such as myocardial infarction, stroke, and cardiovascular mortality.^[33]^ Combining these conditions leads to a higher cardiovascular disease burden and worse outcomes than each condition alone.^[34]^

#### 3.7.2. Impaired cardiac function.

Poorly controlled diabetes can contribute to the development and progression of HF by causing structural and functional changes in the heart. This can lead to reduced cardiac function, increased risk of HF exacerbations, and poorer prognosis.^[35]^

#### 3.7.3. Complications.

HF and diabetes are associated with an increased risk of complications. Patients with this bidirectional relationship may be more prone to microvascular complications (e.g., diabetic nephropathy, retinopathy) and macrovascular complications (e.g., coronary artery disease, peripheral artery disease), which can further impact prognosis.^[20,36]^

#### 3.7.4. Comorbidities.

Comorbidities, such as hypertension, obesity, and chronic kidney disease, can complicate the management of HF and diabetes and contribute to a worse prognosis. Close monitoring and management of these comorbidities are crucial for improving outcomes.^[36]^

#### 3.7.5. Treatment response.

The prognosis can be influenced by the response to treatment. Optimal management of HF with guideline-directed medical therapy, including medications targeting neurohormonal pathways, device therapy (e.g., implantable cardioverter-defibrillators, Cardiac resynchronization therapy), and lifestyle modifications, can improve symptoms, reduce hospitalizations, and prolong survival.^[37]^ Similarly, achieving and maintaining reasonable glycemic control through lifestyle modifications and appropriate use of antihyperglycemic medications can help mitigate complications and improve outcomes.^[38]^

#### 3.7.6. Patient adherence.

Adherence to treatment regimens, including medication adherence, dietary modifications, regular physical activity, and self-monitoring of blood glucose levels, plays a crucial role in determining the prognosis. Patient education, support, and involvement in shared decision-making are essential to promote adherence and improve outcomes.^[39]^

It is important to note that prognosis can vary widely among individuals, and individualized assessment and management are necessary. Regular follow-up, monitoring of symptoms and disease markers, and collaboration between healthcare providers and patients are vital for optimizing prognosis in this population.^[39]^

### 3.8. Complications

The bidirectional relationship between HF and diabetes mellitus can increase the risk of complications. These complications can significantly impact the quality of life, increase healthcare utilization, and worsen outcomes. Here are some critical complications associated with this relationship:

#### 3.8.1. Cardiovascular complications.

Myocardial infarction: Patients with HF and diabetes have a higher risk of experiencing a heart attack due to the combined effects of underlying coronary artery disease, atherosclerosis, and impaired cardiac function.^[23]^

Stroke: The presence of diabetes and HF increases the risk of ischemic and hemorrhagic strokes, often attributed to the vascular complications associated with diabetes and the potential for embolic events in HF patients with atrial fibrillation.^[35]^

Peripheral artery disease: Diabetes and HF can contribute to the development of peripheral artery disease, characterized by reduced blood flow to the extremities, resulting in symptoms such as leg pain, non-healing wounds, and increased risk of limb amputation.^[20]^

#### 3.8.2. Renal complications.

Diabetic nephropathy: Diabetes is a leading cause of chronic kidney disease, and the combination of diabetes and HF further increases the risk of developing diabetic nephropathy, leading to progressive loss of renal function.^[20]^

Fluid and electrolyte imbalance: HF can cause fluid retention, leading to volume overload and edema, which can further strain the kidneys and impair renal function.^[36]^

#### 3.8.3. Metabolic complications:

Diabetic ketoacidosis: In individuals with uncontrolled diabetes, the development of HF can complicate glycemic control and increase the risk of diabetic ketoacidosis, a life-threatening metabolic emergency characterized by elevated ketone levels and acidosis.^[35]^

Hypoglycemia: Some antihyperglycemic medications used in managing diabetes can increase the risk of hypoglycemia, which can be particularly problematic in patients with HF due to the impact on cardiac function and symptoms.^[35]^

#### 3.8.4. Pulmonary complications.

Pulmonary edema: HF can lead to fluid accumulation in the lungs, resulting in pulmonary edema and respiratory distress. This can be further exacerbated by comorbidities such as chronic obstructive pulmonary disease or sleep apnea.^[37]^

#### 3.8.5. Quality of life.

Symptom burden: The combination of HF and diabetes can result in a significant symptom burden, including fatigue, dyspnea, exercise intolerance, polyuria, and polydipsia, impacting daily activities and quality of life.^[20]^

Depression and anxiety: The chronic nature of HF and diabetes, along with the associated complications and lifestyle modifications, can contribute to depression and anxiety disorders, further affecting overall well-being.^[40]^

Early recognition and proactive management of these complications are essential to prevent disease progression, improve outcomes, and enhance the overall quality of life for individuals with the bidirectional relationship between HF and diabetes.^[40]^

## 4. Conclusion

The bidirectional relationship between HF and diabetes mellitus poses significant challenges in clinical management. The coexistence of these 2 conditions amplifies the risk of complications, worsens prognosis, and increases healthcare utilization. Understanding this relationship pathophysiological mechanisms, shared risk factors, and clinical implications is crucial for effective management.

Evidence-based management strategies for HF and diabetes involve a multidisciplinary approach that includes lifestyle modifications, pharmacological interventions, and targeted monitoring. Optimization of glycemic control, blood pressure, and lipid levels, along with guideline-directed medical therapies for HF, plays a pivotal role in improving outcomes.

Emerging therapies, such as sodium-glucose co-transporter-2 inhibitors and glucagon-like peptide-1 receptor agonists, have shown promising results in reducing cardiovascular events, hospitalizations, and mortality in patients with HF and diabetes. These agents offer additional benefits beyond glycemic control, including favorable effects on cardiovascular and renal outcomes.

Collaboration between cardiologists, endocrinologists, primary care physicians, and other healthcare providers is essential for a comprehensive and individualized approach to managing the bidirectional relationship between HF and diabetes. Regular monitoring, patient education, and adherence to treatment plans are crucial to optimize outcomes and improve the overall quality of life for affected individuals.

Future research should focus on identifying novel therapeutic targets, refining risk stratification tools, and exploring personalized management strategies tailored to the unique needs of patients with HF and diabetes. Healthcare providers can significantly reduce this challenging clinical scenario morbidity, mortality, and healthcare burden by addressing the complex interplay between these 2 conditions.

## Author contributions

**Conceptualization:** Chukwuka Elendu, Dependable C. Amaechi, Tochi C. Elendu, Manizha Ashna, Jennifer Ross-Comptis, Sandra O. Ansong, Akinbayo A. Akintunde, Chigozirim M. Agu-ben.

**Data curation:** Chukwuka Elendu, Dependable C. Amaechi, Tochi C. Elendu, Clinton A. Olawuni, Erica I. Pius.

**Formal analysis:** Chukwuka Elendu, Dependable C. Amaechi, Tochi C. Elendu.

**Investigation:** Chukwuka Elendu, Dependable C. Amaechi, Tochi C. Elendu.

**Methodology:** Chukwuka Elendu, Dependable C. Amaechi, Tochi C. Elendu, Sandra O. Ansong, Emmanuel O. Egbunu, Geraldine C. Okafor, Onyekachukwu R. Ekwem.

**Project administration:** Chukwuka Elendu, Dependable C. Amaechi, Tochi C. Elendu.

**Resources:** Chukwuka Elendu, Dependable C. Amaechi, Tochi C. Elendu.

**Software:** Chukwuka Elendu, Dependable C. Amaechi, Tochi C. Elendu.

**Supervision:** Chukwuka Elendu, Dependable C. Amaechi, Tochi C. Elendu, Manizha Ashna.

**Validation:** Chukwuka Elendu, Dependable C. Amaechi, Tochi C. Elendu.

**Visualization:** Chukwuka Elendu, Dependable C. Amaechi, Tochi C. Elendu, Manizha Ashna, Jennifer Ross-Comptis, Akinbayo A. Akintunde, Boluwatife A. Oyedele, Mercy O. Asekhauno, Brandon Carl Monika Pouekoua.

**Writing – original draft:** Chukwuka Elendu, Dependable C. Amaechi, Tochi C. Elendu, Manizha Ashna, Jennifer Ross-Comptis, Mercy O. Asekhauno.

**Writing – review & editing:** Chukwuka Elendu, Dependable C. Amaechi, Tochi C. Elendu, Sandra O. Ansong, Emmanuel O. Egbunu, Klein A. Jingwa, Akinbayo A. Akintunde, Chidinma M. Ogah, Melissa O. Edeko, Ayodeji V. Ibitoye, Mojetoluwa O. Ogunseye, Chisom E. Alakwe-Ojimba, Eunice K. Omeludike, Chibuike A. Oguine, Rechner N. Afuh, Mercy O. Asekhauno, John A. Ladele, Chinonso B. Okoro, Brandon Carl Monika Pouekoua, Joseph S. Adenikinju, Chigozirim M. Agu-ben, Ooreofe Aborisade.
